# A self-report of the Healer’s art by junior doctors: does the course have a lasting influence on personal experience of humanism, self-nurturing skills and medical counterculture?

**DOI:** 10.1186/s12909-019-1877-3

**Published:** 2019-11-29

**Authors:** Chanakya Jaiswal, Katrina Anderson, Emily Haesler

**Affiliations:** 1 Australian National University Medical School, Academic Unit of General Practice, Canberra, Australia; 2 Curtin University, Western Australian Group for Evidence Informed Healthcare Practice: A Joanna Briggs Institute Centre of Excellence, School of Nursing, Midwifery and Paramedicine, Perth, Australia; 3 La Trobe University, School of Nursing and Midwifery, Melbourne, Australia

**Keywords:** Medical education, Humanism, Self-care

## Abstract

**Background:**

Healer’s Art (HA) is a voluntary course offered during medical school. The course aims to address the growing loss of meaning and commitment experienced by doctors through the exploration of compassion, empathy and awe in medicine, and early exposure to a supportive community of practice. This project aimed to evaluate the potential influence of HA on junior doctor graduates.

**Methods:**

Junior doctors who had undertaken HA during their medical studies were interviewed. A thematic analysis was performed on the results of these semi-structured interviews.

**Results:**

Ten junior doctors who had undertaken the HA course participated in interviews. All interviewees described the HA as a positive and enlightening experience in their medical education. The thematic analysis identified four major themes: developing empathy in the doctor-patient journey, self-care and self-awareness, the creation of a supportive community, and coping with the challenging medical culture.

**Conclusions:**

HA provides experiential learning that enables participants to explore humanistic medicine. Self-selected junior doctors recall the course as a positive experience, and perceive themselves to be continuing to employ the techniques from HA in the healthcare setting. The concepts taught in the HA course appear to have a lasting personal impact on some junior doctors, who identify the course as influencing their self-reported positive patient-doctor relationships and supportive relationships with medical peers.

## Background

The Healer’s Art (HA) is a voluntary medical school course with a focus on values, self-care and humanism that is delivered to first and second year students in 113 medical schools in nine countries around the world, but predominantly in the USA [1, 2]. The HA aims to address the growing loss of meaning and commitment experienced by doctors. Doctors around the world are reporting higher levels of burnout, loss of meaning in their work and struggle with the high demands of patient care [3, 4]. In the world of medical education, junior doctors and students report a hospital environment in which respect and caring are devalued by the system as key qualities doctors bring to the doctor-patient relationship [5]. The “hidden curriculum” is an international issue in medicine and often leads students to primarily focus on only medical science while not affirming crucial qualities of being a doctor, such as compassion and empathy [6–8]. The hidden curriculum teaches doctors to suppress their emotional responses. This can result in adoption of ritualized professional identity, loss of idealism, deterioration of integrity and ethics and the unequivocal acceptance of hierarchy [7, 8].

In the Australian context in 2013, one national mental health organisation conducted a National Mental Health Survey of doctors (*N* = 12,252) and medical students (*N* = 1811). The survey found that doctors under 30 years of age had higher levels of burnout and cynicism than any other age range [3]. Cynicism and disenchantment from burnout might translate into poor colleague and patient interactions [3, 4]. Doctors experiencing burnout can lose their values, dignity and spirit, leading to low productivity, diminished patient care, greater likelihood of errors and a loss of empathy [5]. The consequences of burnout for doctors can extend to relationship breakdown, substance abuse, isolation, suicidal ideation and suicide [5]. The impact of doctor burnout has the potential to negatively impact the ways in which care is delivered to patients [3, 4]. The issues associated with doctor burnout have been identified in medical cohorts internationally [9, 10].

The HA course seeks to address some of the issues described above by creating a community counterculture to the hidden curriculum. The course aims to empower and teach medical students self-nurturing skills to insulate them from stress and burnout and provide skills to prevent future burnout [11]. The HA course includes five, three-hour weekly sessions, incorporating experiential learning for both facilitators and students, aiming to illuminate and strengthen participants’ commitment to medicine. Through guided reflections and productive discourse, participants discover and meditate upon the personal values and motivations that led them towards medicine. The HA facilitates discussions in which small groups of students meet with faculty clinicians to explore a range of subjects in a shared personal conversation. Discussion topics include grief, loss, death, spirituality and meaning in medicine. Values of self-care, justice, dedication and compassion are reinforced in these small group discussions. While these principles and values are included in other parts of the medical curriculum, the HA course is unique in the way in which these concepts are presented, the level of self-reflection in which participants engage and the focus given to personal growth. Importantly, the HA is set apart from other parts of the medical curriculum in its use of small groups in which students build close and personal relationships with experienced faculty through the mutual sharing of moments of both vulnerability and strength in the practice of medicine. The course aspires to legitimise open dialogue with colleagues and patients, to reinforce the importance of strong communication skills, to minimise burnout and seeks to build the foundation of a community of practice for students before they enter the medical workforce.

A number of techniques are used to reinforce the HA curriculum over time. The course includes an activity in which participants contribute to a group statement of service, and participants are encouraged to use this statement regularly during personal reflection. Participants are presented with visual and sensory cues to remind them of HA principles, including a “Feely Heart” designed to carry in the pocket during clinical practice and a HA lapel pin to wear in coming years as a way of creating recognition and community amongst alumni.

The Australian National University Medical School (ANUMS) is a graduate medical school that delivers a four-year medical training program. Year cohorts consist of approximately 100 medical students. The majority of graduates enter the Australian Capital Territory (ACT) Health system to complete their internship. Each year around 30 ANUMS students enrol in the voluntary HA program, which is facilitated by 12 to 15 medical staff. The HA faculty, which is recruited from a range of medical disciplines, participate in the course as facilitators on a voluntary basis. At ANUMS, over 30 medical staff and 150 medical school students have completed HA, with many currently working in the ACT Health system.

The aim of the current research was to explore the self-reported impact of HA on junior doctors who have undertaken the course voluntarily during medical school The objectives were to explore junior doctor perceptions of the impact of HA on the way they practice medicine, as well as to explore the potential influence of HA on counterculture in the medical workplace, as perceived by junior doctors.

## Methods

A qualitative analysis of the impact of HA was undertaken, employing purposive sampling, semi-structured interviewing and thematic analysis. The project received ethics approval from the ANU Human Research Ethics Committee and ACT Health Human Research Ethics Committee.

Junior doctors working in the ACT Health system who had undertaken HA while medical students at the ANUMS between 2010 and 2016 were invited via email to register their interest in participating in a semi-structured interview. A purposive sampling template was prepared to ensure selection of an heterogeneous sample including representatives from both genders, a range of ages and at a different career stages.

The semi-structured interviews comprised six questions (Appendix) that were informed by the existing literature [1]. Interviews were conducted by a first year medical student, with all interviews audio recorded and then transcribed by a professional transcribing service. To maintain anonymity of participants, interviews were de-identified in the transcription stage and interviewees were assigned a unique identifier.

All the transcripts were read twice in order to gain a broader understanding of the ideas raised and to check for data saturation. After this, codes that reflected the content and researchers. Next, the codes were applied to the transcripts by two independent researchers using hand coding. Following this, the research team reviewed the coding and discussed discrepancies until consensus was reached. In the next stage of analysis, the initial 11 codes were synthesised into four overarching themes. Finally, the transcripts were reviewed against the overarching themes to check for validity and consistency.

## Results

Ten junior doctors registered interest in the study. These junior doctors represented the demographic diversity outlined in purposive sampling and all were invited to participate in interviews. The selected participants were junior doctors that had completed HA at the ANUMS between four and 6 years previously. The ten individuals ranged in age from 27 to 41 years, with four being male and six being female (Table 1).

**Table 1:** Demographics of participants (*n* = 10)

	n (%)
Age	
25–30	6 (60%)
30–35	3 (30%)
35–40	0 (0%)
41–45	1 (10%)
Sex	
Male	6 (60%)
Female	4 (40%)
Year of Training	
1	3 (30%)
2	6 (60%)
3	0 (0%)
4	1 (10%)

The researchers organised the interview data into 11 codes that represented four key themes (Table 2). Most of the interviewees spoke positively of HA, the principles that were explored in the course and its ongoing impact on the specific doctor over the time. One junior doctor expressed ambivalence towards the experience of HA initially, but throughout the interview this participant described similar themes to other participants, and positive personal impact of the course overall.

**Table 2:**
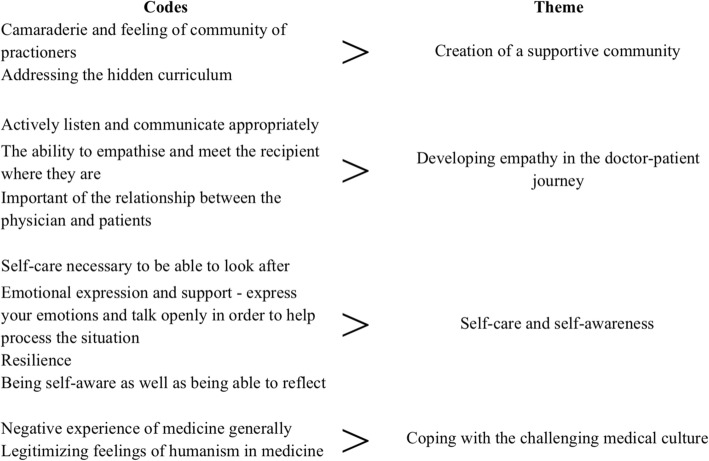
Codes

### Creation of a supportive community

The first theme combined data that identified that the HA course played a significant role in creating a supportive medical community. Participants’ comments reflected their perception that they received support from other doctors who had completed the course. Examples from the junior doctors included:

*I’m in clinical practice, it’s nice to see those people around and connect with those people. (JD4).*

*… we’re trying to have a similar approach to patients and medicine and I think having that connection is really good. (JD4).*

*The fact that you’ve got senior clinicians, as well as other peers of your own who apparently also share these same values, is an important thing to remember. (JD5).*

When discussing the potential of HA having an impact on the wider medical culture, one junior doctor reflected:

*I think looking after each other is, and that is the kind of thing that does actually change the culture of the place. (JD4).*

Junior doctors noted that HA enhanced their feeling of community and allowed inter-collegial bonds to be created:

*I think it went some way towards enhancing my feeling of camaraderie and identifying with the broader medical cohort. (JD5).*

Further, HA aided in connecting junior doctors with a network of humanistic doctors:

*Seeing doctors who are successful in their fields and still cared about the human aspect, so that was huge, (JD1).*

However, despite recognising the network of humanistic doctors who had undertaken HA, one junior doctor (JD1) responded that the HA course had not improved their personal connections and described continuing to feel isolated as a student.

In the interviews participants specifically mentioned the HA lapel pin as a way in which alumni could recognise and connect with those who had completed HA. The junior doctors expressed how the lapel pin reinforced the sense of community:

*For a long time after I’d see the pin on people’s lanyards and realise, hang on this is a bit of a thing, there’s quite a connection that I have with these people, I already know what kind of person they’re going to be if they’re someone who’s been to Healer’s Art. (JD6).*

### Developing empathy in the doctor-patient journey

When asked about a specific situation in which having attended HA helped them to manage a patient, one participant described a clinical encounter that required the application of core HA principles, generous listening as a component of empathy:

*… actually listening to someone for even a few minutes, even in the emergency department, is often enough … I think most of what she needed was someone to talk to. I think that’s something that Healer’s Art deals with. (JD 1).*

The importance of empathy in interactions was highlighted by other junior doctors, for example:

*… one of the principles of Healer’s Art, is meeting people wherever they are [emotionally], I think, and seeing what they need. (JD4).*

Junior doctors also reported that principles of HA had helped them recognise and consider patient priorities when negotiating clinical care:

*Because you’re a doctor, you have your own priorities and things that you need to consider and look out for, but the patient has their own things as well, and that’s a whole other issue. (ID needed here).*

One junior doctor mentioned the journey of empathy from negative feelings towards a patient changing to a more positive one to improve patient care:

*My feeling was the patient should go home and stop bothering us. But I was able to use, use that empathy and that mindfulness to step back from those feelings which were counter-productive … and engage more with what I felt were the underlying concerns which were creating the problem, and was able to more engage with the patient on that basis. (JD5).*

Another junior doctor expressed that HA helped develop their empathetic skills and ability to confront difficult situations:

*Although during med school you do get lessons on how to be empathetic or how to approach difficult situations, I think unless you actually really practice those, which the Healer’s Art program facilitated, you can’t really prepare yourself for the difficult situations. (JD8).*

Another alumnus noted that HA allowed them to better relate to their patient. More than just assisting with development of a richer doctor-patient relationship, they felt that HA allowed them to provide better patient care:

*I think I learnt that from the Healer’s Art, that opening up a little bit, not inappropriately, but attaining a relationship and a rapport, it helps the patient … if you can find something that you can relate to with a patient, it really helps you with that relationship and it helps you know what you need to do for their care. (JD10).*

### Self-care and self-awareness

Physicians caring for themselves while also being self-aware in their medical interactions emerged as a theme. Alumni noted that self-care was an important notion they had learned from HA, and was imperative to being providing patient care:

*I guess I would draw on the principles of self-care, the need to look after myself. If I’m not able to look after myself properly I won’t be able to look after patients. I actually remember one of the doctors who I was in within the small groups, saying that that was one of the lessons that he learned. (JD2).*

*I think one of big things that you learn is you can’t care for anyone else until you’re able care for yourself, and I guess that’s a big thing I took away from the Healer’s Art. (JD3).*

The HA also helped junior doctors recognise both the importance of, and challenges in, being able to practise self-care, and provided them with protective self-care skills when interacting with patients:

*… yes, it’s very hard taking care of patients, and it’s very hard taking care of yourself too, at times. And we can at least look out for each other a little bit and make sure everyone else is doing okay. (JD4).*

*Healer’s Art was also very much about how do you protect yourself, and I think that helps. (JD10).*

The junior doctors who had participated in HA described how the course had increased their self-awareness and practice of reflection, both in practising medicine and in their personal lives:

*Healer’s Art gives people the tools of self-reflection, core values and so forth to better navigate through your own experiences as a doctor. (JD6).*

*Trying to not become too jaded or disillusioned by the challenges that medicine often presents. I think Healer’s Art does that quite well by getting you to reflect on all of the things that make medicine humanistic. (JD9).*

*It [HA] made me realise that it’s not always about me … part of the healing process is to step back and look at how you impact the people around you … not to take over [from patients] and let them have their space to do what they need to do and to talk about what they need to talk about. (JD10).*

### Coping with the challenging medical culture

Participants spontaneously talked about medical culture and the challenges involved as a junior doctor, including working in areas with a bullying culture. An alumnus noted:

*… it’s expected that you do all of these things [as a junior doctor] [but] because you’re so tired a lot of the art [of being a doctor] is lost. (JD1).*

The hospital and medical culture were described at times in strong language:

*There is an inherent hierarchy with a culture that can be quite toxic. (JD9).*

Although a view was expressed that the HA was limited in its ability to influence these specific hospital culture issues, junior doctors recognised that HA principles may positively affect the wider culture of the medical environment:

*I really hope so, and I think particularly relevant at the moment is looking after other junior doctors and that sort of thing, in the media there’s been some terrible mental health things happen. (JD4).*

*In aligning with the principles that the Healer’s Art has, and I hope that in the last few years that I’ve been a doctor that I’ve practised in that way, and by osmosis others around me are seeing how I do things and hopefully agreeing with that approach. And then if I’ve managed to influence anyone, then that would be great. (JD2).*

However, the junior doctors recognised that challenging issues in medical culture are significant, and their address requires more extensive strategies than the HA counterculture:

*Realistically I think that the medical culture’s a ship that’s going to take a long time to turn around. (JD2).*

*I think that there’s a lot of issues with the culture in medicine and I think unfortunately that no one individual can change that”. (JD10).*

*[The culture’s] approach to issues, which is to make people objects, to become compartmentalised and to describe particular issues they might have rather than more holistic concerns of this person. (JD6).*

Despite this, the junior doctors described how the HA course had given them skills to both seek out and provide support to cope with the negative impact of the medical culture:

*What [HA] really encouraged me to do was reach out to my colleagues when they needed it. (JD10).*

*I know that everybody has developed a bit of a vacuum about dealing with patients and what-not, and it’s very well recognised whether you’re coping with the stressful environment. I think looking after each other is, and that is the kind of thing that does actually change the culture of the place. (JD 4).*

*The key set of values which are expressed through Healer’s Art such as patient-centred care or be compassionate, and hopefully that will change the culture of how medicine is practiced which is beneficial both for doctors and people who work in healthcare, as well as for the patients as well. (JD6).*

*I think this program does that [target the toxic culture], hopefully through a bottom up approach. (JD9).*

## Discussion

The HA aims to improve the well-being of medical students/future doctors through experiential learning that seeks to improve communication, self-reflection, meditation and teamwork skills. The course also aims to create a supportive community for students at the start of their careers, ideally maintained over the years as their careers progress. The primary objective of HA is for students and physicians to explore their personal meaning of the practice of medicine. This study has demonstrated that the tenets of HA – the creation of a supportive community counterculture, enhancing self-care and reinforcing the importance of communication and empathy in the doctor-patient journey, are still present in some junior doctors who attended the course numerous years after undertaking HA.

Junior doctors participating in this research suggested that the HA is helping to create a medical community that respects humanism in medicine. Humanism in medicine is when one endeavours to complement medical treatment with compassion, patience and empathy to create a deeper connection with patients [9]. Previous work [1] on HA also reported development of an authentic community amongst medical students. However, the current study is the first time that a supportive community has been described by junior doctors as still present up to 5 years after completion of HA. This research highlighted the role of HA in supporting junior doctors to develop and maintain a supportive community that assists them in coping with the challenging medical culture that in part is due to a “hidden curriculum” [12]. The findings suggested that HA appears to successfully combat this hidden curriculum, which is consistent with previous research on about HA [12]. The HA community can reduce feelings of isolation and provide support and advice in practising patient centred care. The findings of this study are consistent with existing ideas that physicians who are supported are healthier, and that being supported has a therapeutic benefit [13].

Humanism and compassion in medicine are crucial because they are what patients want in their care. These characteristics improve patient care, reduce patient anxiety, and help to optimise a patient’s mood [14]. Compassion in the doctor-patient relationship fosters trust, leading to more accurate and comprehensive diagnoses [15]. The findings of this study are consistent with existing literature that suggests that humanistic doctors provide better healthcare and better health outcomes for both patients and doctors [16–18]. Our results showed that the humanistic ideals that are promoted in HA are maintained by some junior doctors up to 5 years’ after completing the course and these values remained important in their connections with patients.

HA seeks to promote humanism through promoting the importance of the doctor-patient relationship and the practical skills, including communication skills and empathy, through which it can be developed and nurtured. Poor communication is a major problem in the medical community. Between 44,000 and 98,000 deaths per year in the US hospital system may be attributed to poor communication [19]. Patients can be unsatisfied with care delivery despite the doctor believing that the communication is “sufficient” or “excellent” [20]. Further, doctors may overestimate their communication skills. A study of orthopaedic surgeons showed that 75% believed they had effectively communicated their message yet only 21% of their patients agreed [21]. Research has found that patients desire better communication with their doctors [22]. Effective communication between doctor and patient not only promotes trust and support, but can also help patients understand their medical condition and work with the doctor to develop and implement a treatment plan [20, 21, 23].

The HA course includes a focus on developing relationships through empathy, respect and strong communication skills such as active listening. These tools are provided to students in order to promote a connection to both their colleagues and patients on a deeper level. This study showed that these skills that are sharpened in the HA course were described by junior doctors as important skills that they continue to employ in delivering holistic patient care.

Empathy is essential, because understanding patients enables the development of a strong doctor-patient relationship. Empathy allows a doctor to create trust and safety with the patient, which can promote disclosure of necessary diagnostic information, increase patient satisfaction and treatment adherence, improve patient’s quality of life and enhance therapeutic effect [24, 25]. Additionally, empathetic doctors have increased well-being, meaningfulness of work and decreased burnout [24–27]. Empathy may deteriorate throughout medical training, and research has shown its deterioration peaks in the third year, often leading to increased levels of cynicism among medical students as they approach the workforce [25]. Through fostering acceptance of each other, sharing stories and emotions in small groups, and listening to the experiences of senior clinicians, HA encourages students to empathise with their colleagues. By targeting students in their first 2 years of medical training, HA hopes to prevent empathy deterioration that is shown to develop in the later training years [25]. This study showed that junior doctors still recall the empathetic skills learnt in HA and report using these skills in their medical practice. Study participants described how the principles highlighted in the HA course had helped them in their patient interactions and contributed to combating empathy deterioration, which may lead to improved patient care [16].

Emotional burnout (EB) is the feeling of being depleted of one’s emotional resources due to exhaustion from one’s work. It is linked to poor productivity and mood disturbance. A number of studies have identified EB as a significant factor for the junior medical workforce. An Australian study reported that 69% of junior doctors experience EB [28], while a US study identified 45 to 71% of preclinical doctors experienced burnout [29].

The HA teaches self-nurturing skills in order to prevent and mitigate burnout at an early stage. Through meditation, guided and self-reflection, promotion of communication and experiential learning, HA aims to improve emotional intelligence (EI), which is the ability of one to recognise and process their emotions, as well as the emotions of others. Previous research indicates that resident doctors with higher EI and stronger self-nurturing skills experience lower rates of EB and high job satisfaction, and vice versa [30–33]. This study demonstrated that self-selecting junior doctors who had participated in HA displayed self-awareness and self-reflection, and described implementing self-nurturing skills that they were exposed to through the HA course to assist in managing EB.

The culture of medicine has a reputation of being challenging, and this can prevent doctors from receiving support, reducing stress and constructively dealing with disagreements [34]. Also, a toxic culture excuses bullying, harassment and discrimination, problems which are common in the medical workplace. In an Australian study, 49% of Fellows, 54% of trainees and 39% of international medical graduates reported bullying, and 7% reported sexual harassment [34]. In a further study, 74% of second year medical students reported having been taught by humiliation, 58% of students wanting a surgical training path reported experiencing bullying and 30% had experienced sexual abuse [34, 35]. This challenging culture can lead to doctor burnout, loss of empathy, high attrition, increasing doctor suicide and patient deaths [36, 37]. These issues appear to be universal across the medical world.

Our study has shown that courses like the HA could help to protect students and junior doctors negotiating this culture. Junior doctors in our study reported that the concepts and principles highlighted in the HA reminded them of strategies to cope with the challenges of a toxic medical culture. The interviewees identified that the community of practice that is established through the HA course contributes to creating a counterculture in which caring of one’s colleagues is promoted. This finding is consistent with previous HA evaluations that demonstrated that delivery of the HA course created sustained changes in alumni, and that the principles espoused in HA could aid in coping with the stresses and challenges in medicine [1, 2, 11].

This study has a number of limitations. Throughout the medical curriculum, students receive exposure to many of the concepts that are the focus of the HA course. Communication skills, professionalism, compassion and empathy are addressed in various depth in the core curriculum. Therefore, it is possible that the reflections of the junior doctors participating in interviews are not solely the result of participating in the HA. However, the HA curriculum does explore these concepts to a depth not taken by the standard curriculum [1, 2, 11]. Students in our medical school receive no other exposure to the unique experiential learning style and the in-depth exploration of personal experience with experienced faculty that is offered in the HA course. Given the strategies used to reinforce the HA curriculum for participants over time (for example, the lapel pin that was noted by a number of interviewees), it is feasible that the experiences these junior doctors purport to relate to HA are driven by their participation in the course.

The self-selected nature of participation in both this study and the HA elective course are other limitations. It is possible that the findings of this research are biased towards individuals with HA-aligned beliefs who may be more likely to engage in the HA course in their student years. However, our aim was to establish whether the course had impacts into the junior doctor training years and whether, through those participants, the HA may be contributing to cultural change in the workplace. The broad appeal of HA to students and instructors has been previously explored [1]. Additionally, further research by Rabow et al. (2016) has identified that core HA values are imparted on participants, regardless of whether the course is delivered as a voluntary or compulsory component of the medical curriculum [38]. This suggests that the influence of self-selection in HA may not create substantial bias to our findings.

Accessing junior doctors years after their involvement in the HA course proved to be quite challenging, and so there was only a small number of participants in this study. Despite the small sample size, there was an overall consistency of themes suggested that there was saturation of data. One alumnus expressed ambivalent feelings toward some aspects of the HA course and perceived its personal influence to have been minimal, however even this participant identified ways benefit they had been gained from participation. The heterogeneous nature of the sample size further suggests that the findings are generalisable to other participants in the HA course.

Another potential limitation of this study is the influence of the researchers on the research. The interviewer had recently completed the HA course, and this may have influenced both the ways in which the interviews unfolded and the data analysis. Both researchers who undertook the data analysis were significantly involved in the HA. However, the third member of the research team had not completed the HA course, so was positioned to present an outside perspective and confirm the validity of the thematic structure.

An important strength of this study was the ability to study the HA delivered in the same medical school over 9 years, and the potential influence on the medical culture of up to 30% of the junior doctor workforce in one hospital having undertaken the course. The findings suggested that slow changes are unfolding through increasing the focus on humanism in medicine and introduction of a counterculture. However, further research is required to determine the extent of any cultural change, the contribution HA makes to this change and whether the outcomes extend to benefits to the patient experience. Future research in this field could explore the experiences of junior doctors who have not undertaken the HA course or similar courses that use experiential learning and interpersonal faculty relationships to impart humanism, self-nurturing skills and a counterculture.

## Conclusion

This investigation is the first to examine perceptions of the influence of HA in a cohort of junior doctors that had undertaken the course numerous years earlier. The research identified that the HA has a lasting personal influence on the participants engaged in this research, and the course principles were recalled and applied by these junior doctors up to 5 years following course completion. The humanistic principles of HA are grounded in creating a supportive doctor community and helping doctors to cope with the challenging medical culture. The HA was described as positively contributing to ways in which it’s a self-selected group of alumni practice medicine. Medical schools could consider auditing their curriculum to ensure the important concepts delivered in the HA are covered in sufficient depth within the core medical curriculum. Offering the Healer’s Art or similarly focused experiential learning and relationship building courses as a core or elective component of the medical curriculum offers one strategy to promote humanism and self-nurturing skills and to develop countercultures to the hidden curriculum. Junior doctor training programs could explore reasons why current curriculum may have failed to adequately address these issues, and how the core principles of humanism and self-nurturing could be kept alive in the hearts of junior doctors.

## Data Availability

The data that support the findings of this study are available on request from KA. The data is not publicly available due to the data containing information that could compromise research participant privacy.

## Appendix

### Interview Proforma

•How has the Healer’s Art course and its principles contributed to your broad experience of being a doctor in a positive or negative way or not at all?

•How has the Healer’s Art affected or contributed to your ability to provide patient care as a healer in a positive or negative way or not at all?

•Which parts or lessons learnt during The Healer’s Art do you still find resonate the most for you when interacting with your patients?

•Describe a specific situation in which the principles of the Healer’s Art contributed to the way you managed a patient or performed as a doctor.

•Do you feel that your experience of the Healer’s Art is having a wider effect on the culture of the medical environment through you? How?

•How has the Healer’s Art course and its principles enhanced your life personally?
